# Impact of Pea Protein–Pectin Emulsion Gels Containing Soybean Oil on the Whipping Performance of Non-Dairy Whipping Cream

**DOI:** 10.3390/foods15162929

**Published:** 2026-08-21

**Authors:** Ruyue Yin, Qingqing Cao, Keqing Mao, Pei Wang, Fusheng Chen, Lifen Zhang, Xianzhong Zhang

**Affiliations:** 1 College of Food Science and Engineering, Henan University of Technology, Zhengzhou 450001, China; 18231137848@163.com (R.Y.); 15639439385@163.com (Q.C.); 18438636402@163.com (K.M.); fushengc@haut.edu.cn (F.C.); 2 Zhejiang University Zhongyuan Institute, Zhengzhou 450001, China; 15039097631@163.com

**Keywords:** non-dairy whipping cream, pea protein, high-methoxyl pectin, emulsion gel, fat substitute

## Abstract

A non-dairy whipping cream was developed by partially replacing cocoa butter substitute with pea protein–high-methoxyl pectin (PP–HMP) emulsion gel containing soybean oil. The effects of substitution levels (10%, 20%, 30%, 40%, and 50%) on the properties of the pre-whipping cream emulsion and the resulting whipped cream were systematically investigated and compared with those of commercial whipped creams. The properties of the pre-whipping emulsion (apparent viscosity and particle size) and whipped cream (hardness, adhesiveness, elastic modulus, and thixotropic loop area) degraded with increasing PP–HMP emulsion gel substitution levels. Overrun and partial coalescence of fat reached their highest values at the 20% substitution level, at which point the whipped cream also exhibited a uniform microstructure and excellent shape retention. The overrun, hardness, and foam stability of non-dairy whipping cream containing 20% PP–HMP emulsion gel were comparable to those of the commercial whipped cream. These findings demonstrate the potential of PP–HMP emulsion gel as a fat substitute for developing non-dairy whipping cream with reduced hydrogenated fat content.

## 1. Introduction

Non-dairy whipping cream is a vegetable oil-in-water emulsion comprising non-dairy fats, water, sugars, emulsifiers, and stabilizers. Most commercially available products contain hydrogenated palm kernel oil, which is rich in saturated fatty acids [1]. Excessive intake of saturated fatty acids has been associated with obesity, as well as cardiovascular and cerebrovascular diseases [2]. Therefore, the development of fat substitutes for non-dairy whipping cream has garnered considerable research attention.

Recent studies have explored the use of emulsion gels as fat substitutes in food products such as whipping cream. Emulsion gels are edible semisolid systems wherein oil droplets are encapsulated within a three-dimensional gel network [3]. Owing to their high stability, elastic texture, and well-defined microstructure, emulsion gels can mimic the textural properties of saturated fatty acids while reducing their content in food products [4]. In particular, protein-based emulsion gels exhibit excellent textural properties, favorable rheological behavior, and superior storage stability [5].

Pea protein (PP) is characterized by a balanced amino acid composition, low allergenicity, and high nutritional value. However, its low surface charge and low disulfide bond content result in weak gels with poor elasticity, thereby restricting its application in the food industry. These gelation-related limitations can be effectively addressed by blending PP with polysaccharides. For instance, Kamer [6] reported that emulsion gels prepared using 2% PP, 1% guar gum, and 60% olive oil exhibited excellent textural and rheological properties and that these gels can serve as suitable alternatives to solid fats. Electrostatic interactions and hydrogen bonding between pectin and PP promote hydrogen bond formation and hydrophobic interactions within the PP gel network, thereby enhancing gel strength [7]. This indicates that pectin can serve as a gel enhancer for PP.

The oil phase influences droplet size, interfacial characteristics, and network strength of emulsion gels, thereby affecting their functionality as fat substitutes in plant-based cream [8]. Highly polar oils weaken oil–protein hydrophobic interactions and promote protein–protein interactions, resulting in strong gel networks [9]. In addition, fat crystallization influences the partial coalescence of fat and ultimately the whipping and foaming properties of cream [10]. The quality of cream is also determined by the amount of emulsion gels used as a fat substitute. For instance, replacing 30% or 60% of hydrogenated palm kernel oil with beeswax oleo gel improved elasticity, overrun, firmness, and bubble homogeneity, whereas 90% substitution caused a considerable reduction in overrun and foam collapse [11]. Increasing the oil volume fraction promoted crystallization and strengthened the three-dimensional crystal network in non-dairy whipping cream. Replacing 20% of hydrogenated palm kernel oil with soybean oil bodies considerably reduced particle size and interfacial tension while increasing apparent viscosity and elastic modulus. These changes in turn improved emulsification stability, foam stability, and sensory properties of non-dairy whipping cream, resulting in the highest overall acceptability score [12]. These findings indicate that emulsion gels with an optimal oil phase composition can serve as effective fat substitutes and improve the stability and sensory characteristics of non-dairy whipping cream. Thus, optimizing the oil phase of fat substitutes is an effective method for improving the overall quality of non-dairy whipping cream [13].

Our preliminary studies demonstrated that PP–high-methoxyl pectin (PP–HMP) emulsion gels containing soybean oil are promising candidates for partially replacing 20% hydrogenated oil in non-dairy whipping cream [8]. However, excessive substitution with alternative fat substitutes can severely deteriorate whipping performance and structural stability of cream [11]. Therefore, identifying an optimal substitution level is essential to maintain desirable product quality. In this study, non-dairy whipping cream formulations were prepared by replacing 10–50% of cocoa butter substitute with PP–HMP emulsion gel containing soybean oil. The effects of substitution level on the rheological, textural, microstructural, and whipping properties of non-dairy whipping cream were investigated and compared with those of a commercial whipping cream. The aim was to develop a non-dairy whipping cream with low hydrogenated fat content.

## 2. Materials and Methods

### 2.1. Materials

PP (protein content, 80.98%) was supplied by YuWang Group (Dezhou, Shandong, China). HMP (galacturonic acid content, 75.1%; degree of esterification, 61.0%) was purchased from Macklin (Shanghai, China). Transglutaminase (TG, 120 U/g) was obtained from Dongsheng Biotech (Taixing) Co., Ltd. (Taixing, Jiangsu, China). Cocoa butter substitute (BL-41) was obtained from Yihai Kerry Southseas Oils & Fats Industry (Chiwan) Co., Ltd. (Shenzhen, China). Soybean oil was purchased from a local supermarket in Henan, China. Sodium caseinate, sucrose fatty acid ester (HLB 11), glucose, and other ingredients used in non-dairy whipping cream formulation were of food-grade quality and purchased from Jiahe Xuri Food Additives Company (Zhengzhou, Henan, China). All other chemicals were obtained from Tianjin Kemiou Chemical Reagent Co., Ltd. (Tianjin, China).

### 2.2. Preparation of Non-Dairy Whipping Cream

PP (5 g) was added to 100 mL of distilled water and stirred with a magnetic stirrer (85-2, 0–2400 rpm, Changzhou Jintan Huafeng Instrument Co., Ltd. (Changzhou, Jiangsu, China), and the button was set to the medium rotational speed position at room temperature overnight to obtain a homogeneous dispersion. HMP (0.3 g) was added to the PP suspension and stirred under the same conditions described above for 4 h. The pH of the mixture was adjusted to 6.0 using 1 mol/L HCl and stirred for 1 h to obtain the aqueous phase. Soybean oil at a volume fraction of 20% was used as the oil phase. The aqueous and oil phases were homogenized in a high-shear dispersing emulsifier (FM-200A, FLUKO, Shanghai, China) at 13,000 rpm for 2 min. To catalyze protein crosslinking and strengthen the protein network, the resulting emulsion was treated with TG (30 U/g PP) at 40 °C for 2 h and heated at 85 °C for 30 min. After storage at 4 °C overnight, PP–HMP emulsion gels (hardness: 63.04 ± 2.02 g; water holding capacity: 64.13% ± 2.78%; oil holding capacity: 98.59% ± 0.16%) were obtained [14].

The composition of the non-dairy whipping cream was according to Chen et al. [8]. A pre-whipping cream emulsion was prepared by mixing an oil phase (20.90%) containing 20% cocoa butter substitute, 0.50% sodium caseinate, 0.15% sucrose fatty acid ester, 0.10% Span 60, 0.12% xanthan gum, and 0.03% guar gum with an aqueous phase (79.10%) containing 18% glucose, 10% maltose syrup, 0.06% disodium hydrogen phosphate, 0.04% sodium dihydrogen phosphate, 0.30% sodium chloride, 2.50% granulated sugar, 0.05% vanillin, and 48.15% water at 13,500 rpm for 2 min. The resulting emulsion was homogenized twice using a high-pressure homogenizer (APV 2000, SPX Flow, Rendsburg, Germany) at 50 MPa. The samples were immediately cooled and stored at –18 °C for 24 h. PP–HMP emulsion gel replaced 10%, 20%, 30%, 40%, and 50% of the cocoa butter substitute. The formulation without PP–HMP emulsion gel served as the control. Before whipping, the emulsions were thawed at 4 °C for 12 h [8]. All experiments were performed in triplicate. The non-dairy whipping cream formulation complies with the industry standard SB/T 10419-2017 (Non-dairy whipping cream) [15].

### 2.3. Preparation of Pre-Whipping Cream Emulsions

#### 2.3.1. Apparent Viscosity Analysis

The apparent viscosity of the pre-whipping cream emulsions was measured using an HR10 rheometer (TA Instruments, New Castle, DE, USA) equipped with a 40 mm parallel-plate geometry and a 0.5 mm gap at 25 °C. The shear rate ranged from 0.1 to 100 s^−1^, and the apparent viscosity was recorded as a function of shear rate [16]. Shear stress (*τ*)–shear rate (*γ*) data were fitted to the following Herschel–Bulkley model [17]:

(1) $$\tau ={\tau_{0}+K(\gamma )}^{n}$$

#### 2.3.2. Particle Size and Zeta Potential Analysis

The particle size and zeta potential were determined using a nanoparticle size analyzer (BeNano 90, Dandong Bettersize Instruments Ltd., Dandong, Liaoning, China). Prior to analysis, the emulsion was diluted with distilled water. Refractive indices of 1.59 and 1.33 were used for the dispersed phase and the aqueous medium, respectively. All measurements were performed at 25 °C.

### 2.4. Characteristics Analysis of Non-Dairy Whipping Cream

#### 2.4.1. Determination of Partial Coalescence of Fat

The partial coalescence of fat was analyzed using a method reported by Chen et al. [18]. Whipped cream (20 g) was mixed with 10 g of Oil Red O solution prepared by dissolving 0.01 g of Oil Red O in 1000 g of corn oil and centrifuged at 10,000 rpm for 40 min. The upper clear red oil layer was collected, and its absorbance was measured at 520 nm using corn oil as the blank. The partial coalescence of fat (Φ_d_, %) was calculated using Equation (1).

(2) Φ_d_ = m_1_(α – 1)/m_2_φ,

where Φ_d_ is the partial coalescence of fat (%), φ is the fat mass fraction in the emulsion, m_1_ is the mass of the Oil Red O solution, m_2_ is the mass of the whipped cream sample, and α is the absorbance ratio of the dye solution before and after centrifugation.

#### 2.4.2. Overrun, Whipping Time, and Foam Collapse Rate

Frozen cream emulsions were thawed at 4 °C for 12 h prior to whipping. Samples were whipped using an electric mixer (S-LD500, Joyoung, Jinan, Shandong, China) at a speed setting of 3 until firm peaks formed. The time at which the cream was obtained and broke away from the wires and bowl was defined as the whipping time. The whipped cream was transferred to a piping bag and extruded onto a flat surface at room temperature (25 ± 2 °C). The height of the extruded cream was measured immediately (H_1_) and again after 2 h (H_2_) of storage at room temperature (25 ± 2 °C). The overrun and foam collapse rates of the non-dairy whipping cream were calculated as follows [16]:

(3) Overrun (%) = (W_1_ – W_2_)/W_2_ × 100,

(4) Foam collapse rate (%) = (H_1_ – H_2_)/H_1_ × 100,

where W_1_ and W_2_ are the weights (g) of the pre-whipping cream emulsion and whipped cream, respectively, at the same volume, and H_1_ and H_2_ are the heights (cm) of whipped cream peaks at 0 h and after 2 h of standing, respectively.

#### 2.4.3. Texture Profile Analysis

For texture profile analysis using TA.XT Plus (Stable Micro Systems Ltd., Surrey, UK), the samples were placed in aluminum containers (50 mm × 30 mm). Measurements were conducted using a P25 probe with a trigger force of 1 g, a test speed of 1.0 mm/s, and a compression distance of 20 mm [16]. Texture was characterized in terms of hardness and adhesiveness, and all measurements were performed in triplicate.

#### 2.4.4. Analysis of Rheological Properties

Rheological measurements were performed using an HR10 rheometer (TA Instruments, New Castle, DE, USA) equipped with a 40 mm parallel-plate geometry and a 0.5 mm gap. The linear viscoelastic region was determined via strain sweep measurements. Frequency sweeps were conducted over 0.1–100 Hz at 1% strain, 5 s intervals, and 25 °C [19].

The thixotropic behavior of the non-dairy whipping cream was evaluated by increasing the shear rate logarithmically from 0.01 to 100 s^−1^ over 180 s (upward ramp), followed immediately by a decreasing ramp from 100 to 0.01 s^−1^ over 180 s (downward ramp). The thixotropic loop area, i.e., the area enclosed between the upward and downward flow curves, was calculated to quantify the energy required to disrupt the internal network structure. All tests were performed on fresh whipped cream samples.

#### 2.4.5. Optical Microscopy

The microstructure of the whipped cream was observed using an optical microscope (ML31, Mshot, Guangzhou, China) equipped with a 10× objective lens [20]. Samples were carefully placed on glass slides and covered with coverslips before observation.

### 2.5. Comparison Analysis of Commercial Whipped Cream

Two commercial whipped creams, designated as commercial whipped creams 1 (main component: water, hydrogenated palm kernel oil, glucose, glucose syrup, granulated sugar; fat content, 16.7 g/100 g) and 2 (main component: water, hydrogenated vegetable oil, glucose, maltose syrup, granulated sugar; fat content, 14.4 g/100 g), were selected for comparison with the non-dairy whipping cream containing PP–HMP emulsion gel. The compositions of the commercial whipped creams are detailed in Table S1. All measurements were conducted using the methods described above.

### 2.6. Statistical Analyses

All experiments were conducted in triplicate. Graphical representations were created using Origin 2021 software. Statistical analyses were performed using IBM SPSS Statistics Version 21.0. Duncan’s multiple range test was applied to identify significant differences at a confidence level of *p* < 0.05.

## 3. Results and Discussion

### 3.1. Properties of Pre-Whipping Cream Emulsions

#### 3.1.1. Apparent Viscosity

**Figure 1:**
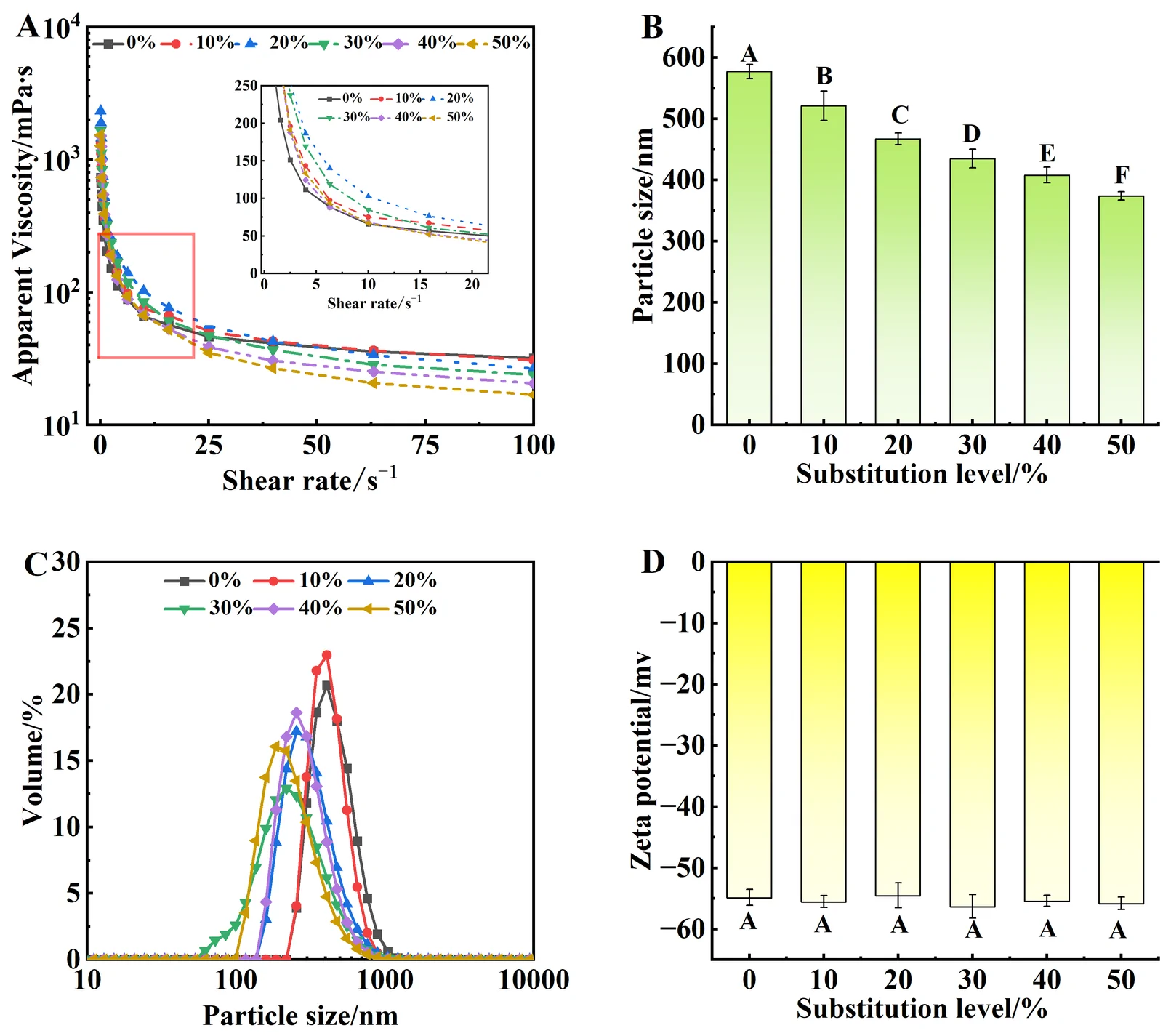
Effects of substitution level of PP-HMP emulsion gel on the apparent viscosity (**A**), particle size (**B**), particle size distribution (**C**), and zeta potential (**D**) of pre-whipping cream emulsion. Different letters in the bar charts indicate significant differences (*p* < 0.05).

Apparent viscosity is a key parameter for characterizing the pre-whipping cream emulsions [19]. As shown in Figure 1A, the apparent viscosity decreased with increasing shear rates, revealing shear-thinning behavior. When the PP–HMP emulsion-gel substitution level exceeded 30%, the apparent viscosity decreased progressively with further increases in substitution level, revealing a nonmonotonic dependence on substitution level. The decrease in viscosity at high substitution levels can be attributed to the reduction in solid fat content and consequent weakening of interactions among fat globules in the emulsion. Meanwhile, lower viscosity reduces the frequency of collisions between fat globules, thereby contributing to the storage stability of the pre-whipping cream emulsion [21]. An ideal whipping cream must appropriately balance emulsion stability and whipping ease [22]. At moderate substitution levels, sufficient fat globules remain to stabilize the emulsion and facilitate adequate partial coalescence during whipping without compromising foam stability and the overall product quality [18]. Qian et al. [12] reported that substituting 20% of hydrogenated palm kernel oil with soybean oil bodies optimally reduced particle size and interfacial tension while increasing apparent viscosity and elastic modulus, thereby improving emulsification stability and foam stability. In this study, the apparent viscosity of the pre-whipping cream emulsion substituted with 20% PP–HMP emulsion gel matched or exceeded that of the control across the entire shear rate range, again reflecting the improved whipping properties of the emulsion.

The fitted Herschel–Bulkley parameters are summarized in Table S2. The yield stress (*τ*_0_), representing the minimum stress required to initiate flow, increased from 0.134 Pa at 0% substitution to a maximum of 0.304 Pa at 20% substitution, indicating that the optimal substitution level (20%) maximally strengthens the internal network structure. This enhancement is likely attributable to the appropriate balance between the fat globule network and the protein–polysaccharide interactions that reinforce the structural integrity of the emulsion [23]. The consistency coefficient (*K*), reflecting the apparent “thickness” of the system, reached its peak (0.171 Pa·s*^n^*) at 20% substitution. This trend suggests that a 20% emulsion gel substitution level effectively increases the flow resistance of the cream, thereby improving its body and mouthfeel [17]. The flow behavior index (*n*) of the samples ranged from 0.579 to 0.764, confirming the characteristic shear-thinning (pseudoplastic) behavior of the cream. Notably, *n* was minimized (0.579) at 20% substitution, indicating the highest degree of shear sensitivity at this substitution level. Collectively, these results demonstrate that 20% substitution optimizes the balance between structural strength and shear-thinning behavior, which is critical for enhancing the whipping properties and foam stability in non-dairy whipping creams [8].

#### 3.1.2. Particle Size and Zeta Potential

Particle size and distribution are important indicators of emulsion stability and can considerably influence the whipping properties of cream emulsions [24]. As shown in Figure 1B, increasing the PP–HMP emulsion gel substitution level considerably decreased the particle size of the pre-whipping cream emulsions. All samples exhibited a unimodal particle size distribution within 100–1000 nm, and the distribution peak gradually shifted toward smaller particle sizes with increasing substitution level (Figure 1C). The unimodal distribution indicates satisfactory emulsifying performance of the emulsion gel. The leftward shift in the peak suggests that with increasing substitution level, the particle size of the dispersed phase decreased [25]. Fu et al. [26] reported that whipping cream emulsions with smaller and more homogeneous particle size distributions exhibited shorter whipping times and higher overrun. No significant differences in zeta potential were observed among the formulations despite the increase in PP–HMP emulsion gel substitution level (Figure 1D). The zeta potentials of all samples exceeded 50 mV, well above the generally recognized threshold (30 mV) for electrostatically stabilized emulsions. Therefore, the emulsion was inferred to be stabilized through strong electrostatic repulsion among the oil droplets [27].

### 3.2. Characteristics of Non-Dairy Whipping Cream

#### 3.2.1. Partial Coalescence of Fat

Partial coalescence refers to the irreversible aggregation of fat globules mediated by interactions between solid fat crystals and liquid fat [28]. Table 1 shows the effects of PP–HMP emulsion gel substitution level on the partial coalescence of fat in non-dairy whipping cream. With increasing PP–HMP emulsion gel substitution level, the partial coalescence degree first increased and then decreased; however, all substituted samples exhibited higher values than the control. The highest measured partial coalescence degree of 48.83% was observed at 20% substitution, which was considerably higher than that of the control (37.18%) (Table 1). Moderate substitution levels reduced the apparent viscosity of the pre-whipping cream emulsion, thereby facilitating air incorporation during whipping and promoting collisions among fat globules, which favored partial coalescence [29]. However, further increases in substitution levels reduced the concentration of crystallizable fat globules in the dispersed system. Consequently, the probability of collisions among fat globules under shear stress decreased, and fat crystals became less likely to penetrate the interfacial films and form bridges between adjacent fat globules. This ultimately resulted in a decreased partial coalescence degree [30].

#### 3.2.2. Whipping Time, Overrun, and Foam Collapse Rate

Table 1 shows the effects of PP–HMP emulsion gel substitution level on the whipping characteristics of non-dairy whipping cream. Whipping time considerably increased with increasing substitution level. At a substitution level of ≤30%, no significant difference in overrun was observed in the formulations compared with the control. The increase in overrun at moderate substitution level can be attributed to the enhanced partial coalescence of fat globules, which promoted the formation of a stable foam structure [31]. However, overrun decreased when the substitution level exceeded 30%. This is because some proteins on the surface of fat globules could not be competitively adsorbed by the emulsifier, thereby hindering effective partial coalescence and air incorporation during whipping [16].

**Figure 2:**
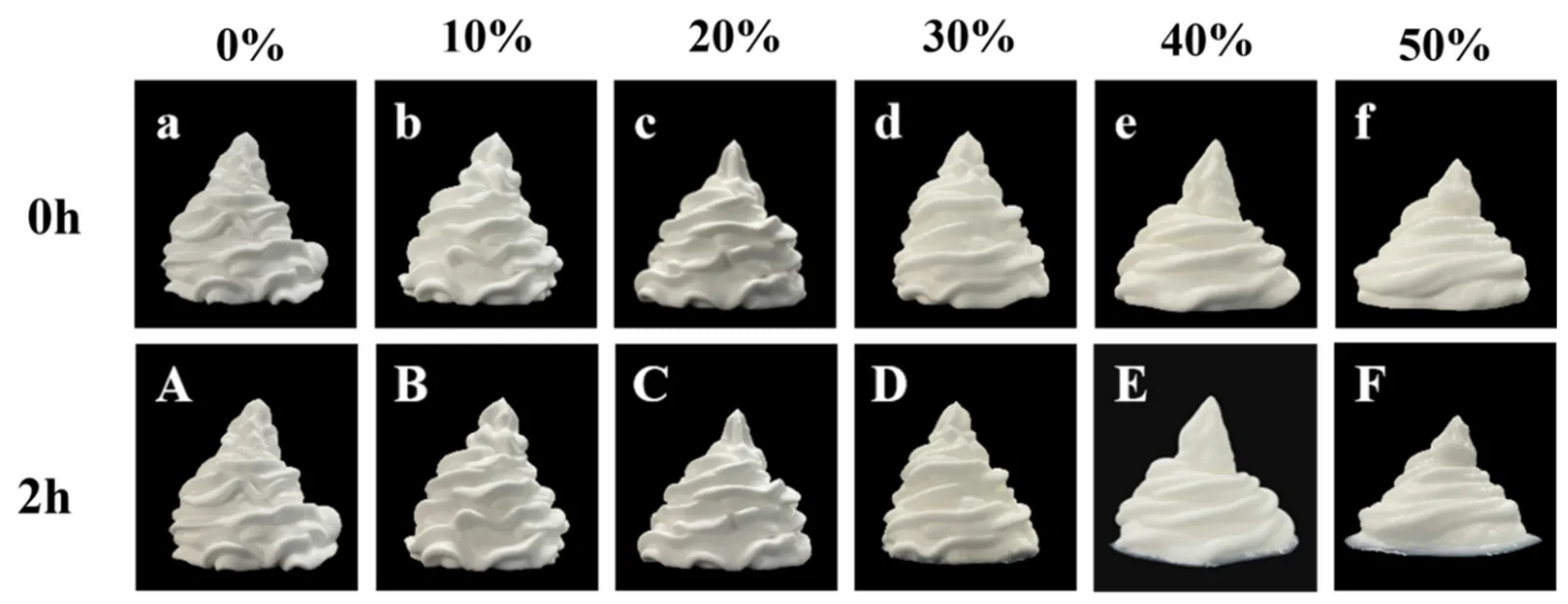
Appearance of whipped creams immediately after whipping (0 h) (**a**–**f**) and after 2 h of storage (**A**–**F**).

Figure 2 shows the appearance of the non-dairy whipping cream both immediately after whipping and after 2 h of storage. Samples containing 30% or less PP–HMP emulsion gel exhibited sharper edges and a more uniform internal structure. In contrast, samples containing 40% or more emulsion gel exhibited partial collapse, insufficient firmness, and a coarse cross-sectional structure. After 2 h of storage, all samples exhibited varying degrees of collapse (Figure 2), and the foam collapse rates ranged from 4.97% to 10.60% (Table 1). Higher substitution levels reduced the total fat content in the pre-whipping cream emulsion, thereby reducing the partial coalescence of fat globules. Consequently, the crystalline network became insufficient to maintain the integrity of the encapsulated air bubbles, resulting in higher foam collapse rates [26].

**Figure 3:**
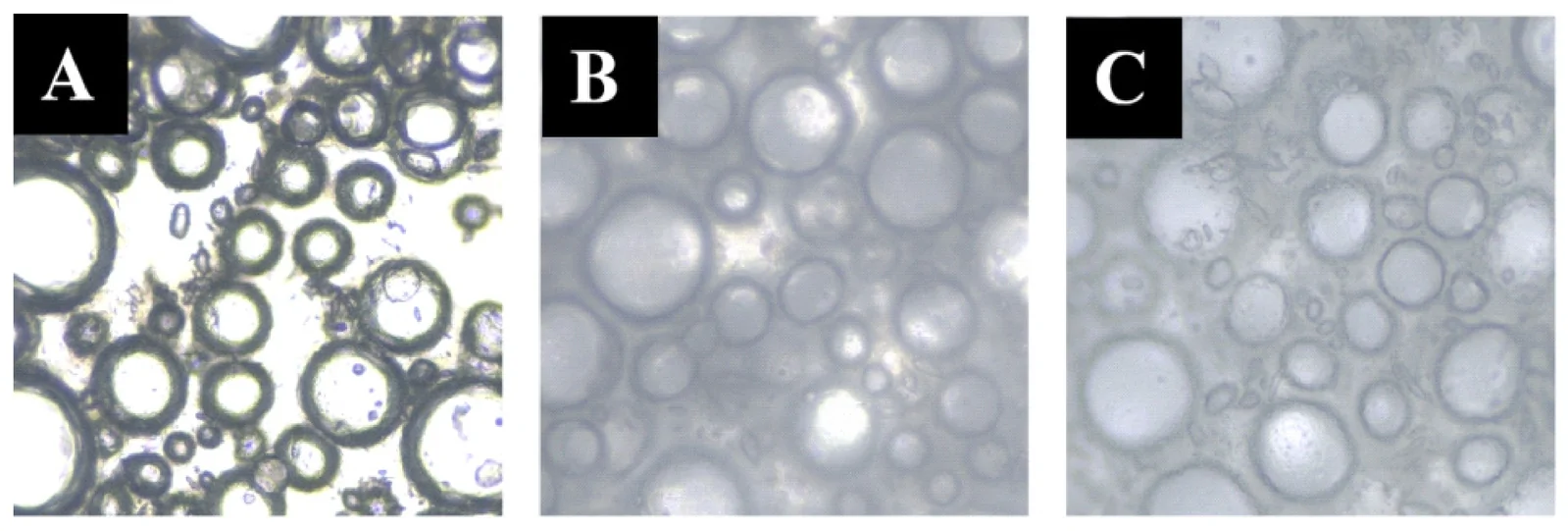

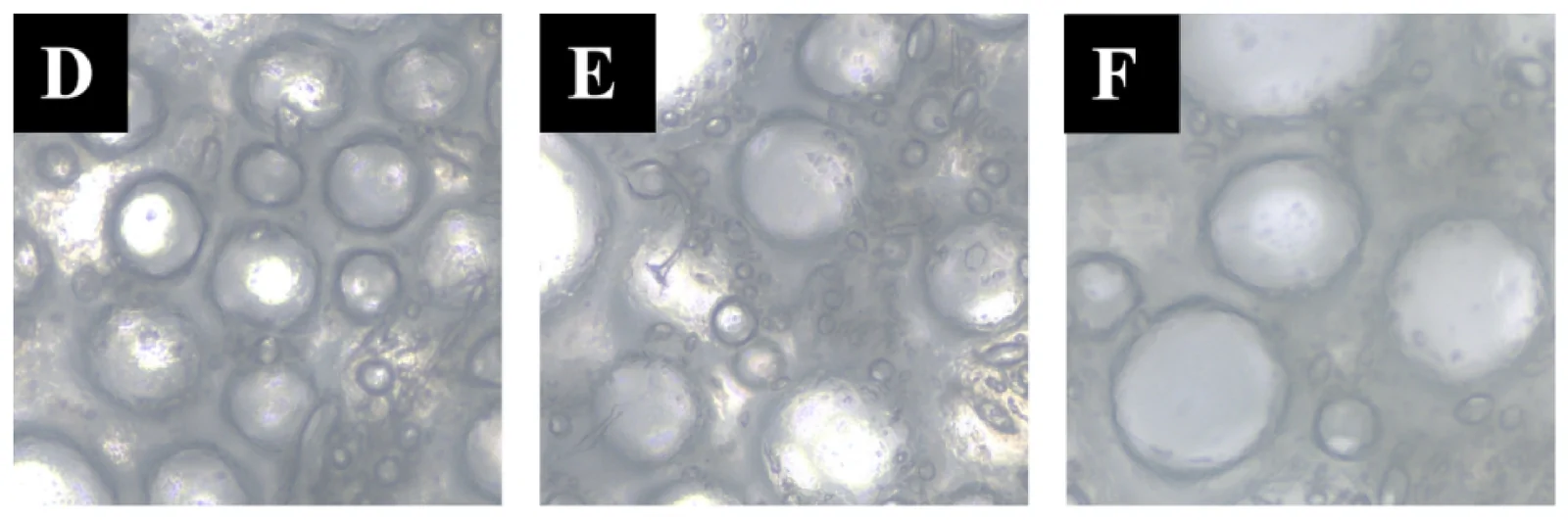
Optical micrographs of whipped creams with different PP–HMP emulsion gel substitution levels: (**A**) 0%, (**B**) 10%, (**C**) 20%, (**D**) 30%, (**E**) 40%, and (**F**) 50%. Images were obtained under a ×10 objective lens.

The bubble size (observed via optical microscopy) provides critical insights into the foam structure and stability of whipped cream. Figure 3 shows the microstructures of the whipped cream, indicating the effect of substitution level on air bubble formation. The control sample exhibited smaller and more uniformly distributed air bubbles. As the substitution level increased, the proportion of large bubbles gradually increased. At 10–30% substitution, the whipped cream still contained smaller and more uniformly distributed bubbles. Smaller, more uniformly distributed bubbles generally indicate higher overrun and better shape retention than larger bubbles, which are susceptible to disproportionation and coalescence, leading to foam collapse [32]. The bubble-size distribution also reflects the partial coalescence of fat during whipping. More specifically, a higher partial coalescence of fat effectively stabilizes small bubbles against coalescence [33]. The increase in bubble size with increasing PP–HMP emulsion gel substitution level (Figure 3) indicates a weaker fat crystal network that could not stabilize the incorporated air bubbles, consistent with the reduced hardness and increased foam collapse rate at substitution levels above 30%.

#### 3.2.3. Textural Properties

**Figure 4:**
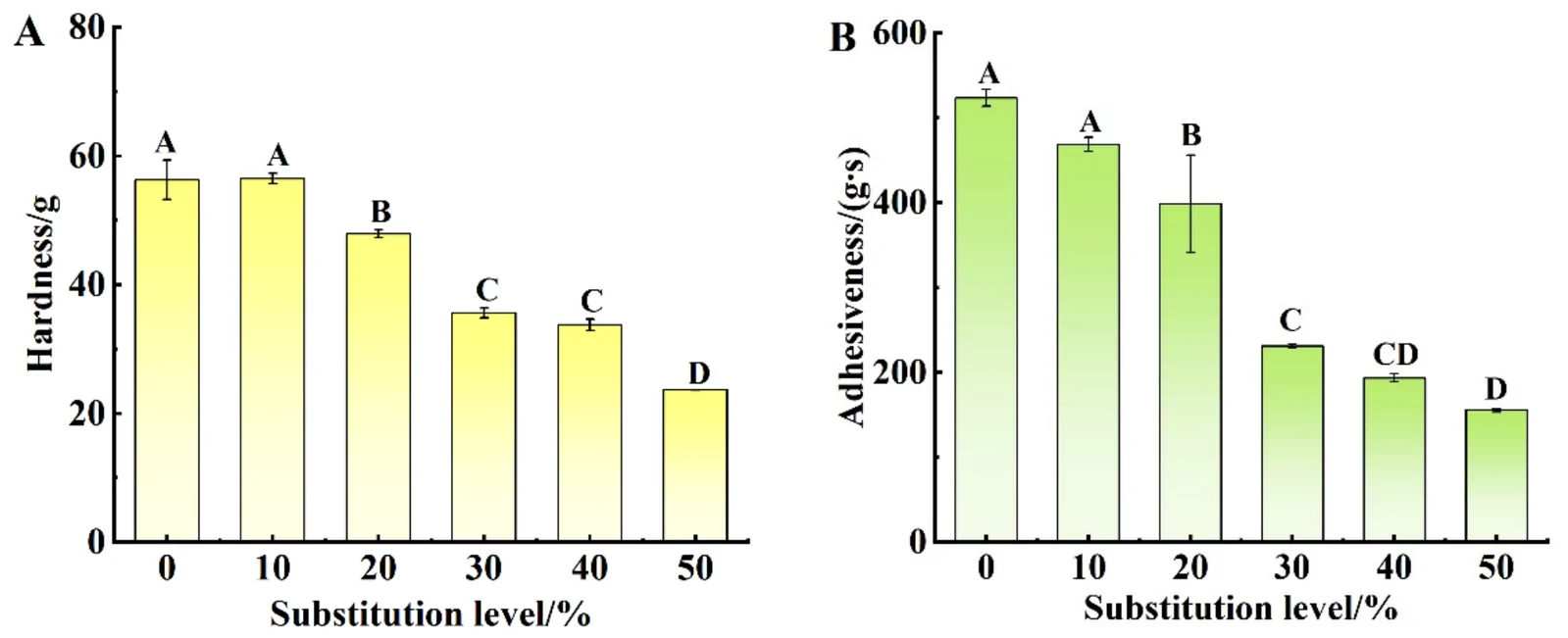
Effects of PP–HMP emulsion gel substitution level on the (**A**) hardness and (**B**) adhesiveness of whipped cream. Different letters indicate significant differences (*p* < 0.05).

Figure 4 shows the effects of PP–HMP emulsion gel substitution level on the textural properties of whipped cream. The hardness and adhesiveness were reduced at substitution levels above 20%. A drastic decrease was observed between 20% and 30%, whereas no significant decrease was observed between 30% and 40%. This reduction can be attributed to the decrease in the amount of crystallizable fat globules available for partial coalescence and network formation. In addition, the lower fat content increased the concentration of proteins and emulsifiers adsorbed on the fat globules, leading to the formation of a thicker interfacial layer. Such an interfacial layer acts as a stronger physical barrier, reducing collisions and partial coalescence among fat globules under shear stress during whipping [18,34]. Consequently, the formation of a rigid, three-dimensional fat crystal network that supports the foam structure was hindered, leading to considerably lower hardness and adhesiveness of the whipped cream [35]. Consistent with the observations in Figure 2, the reductions in hardness and adhesiveness became particularly pronounced when the substitution level was between 20% and 30%; this indicates that the foam exhibited insufficient structural strength for food processing applications that require good whipping performance and shape retention [18]. Du and Meng [36] reported that adding oleogels as fat substitutes weakens the network structure of ice cream, thereby reducing its hardness.

#### 3.2.4. Rheological Properties

**Figure 5:**
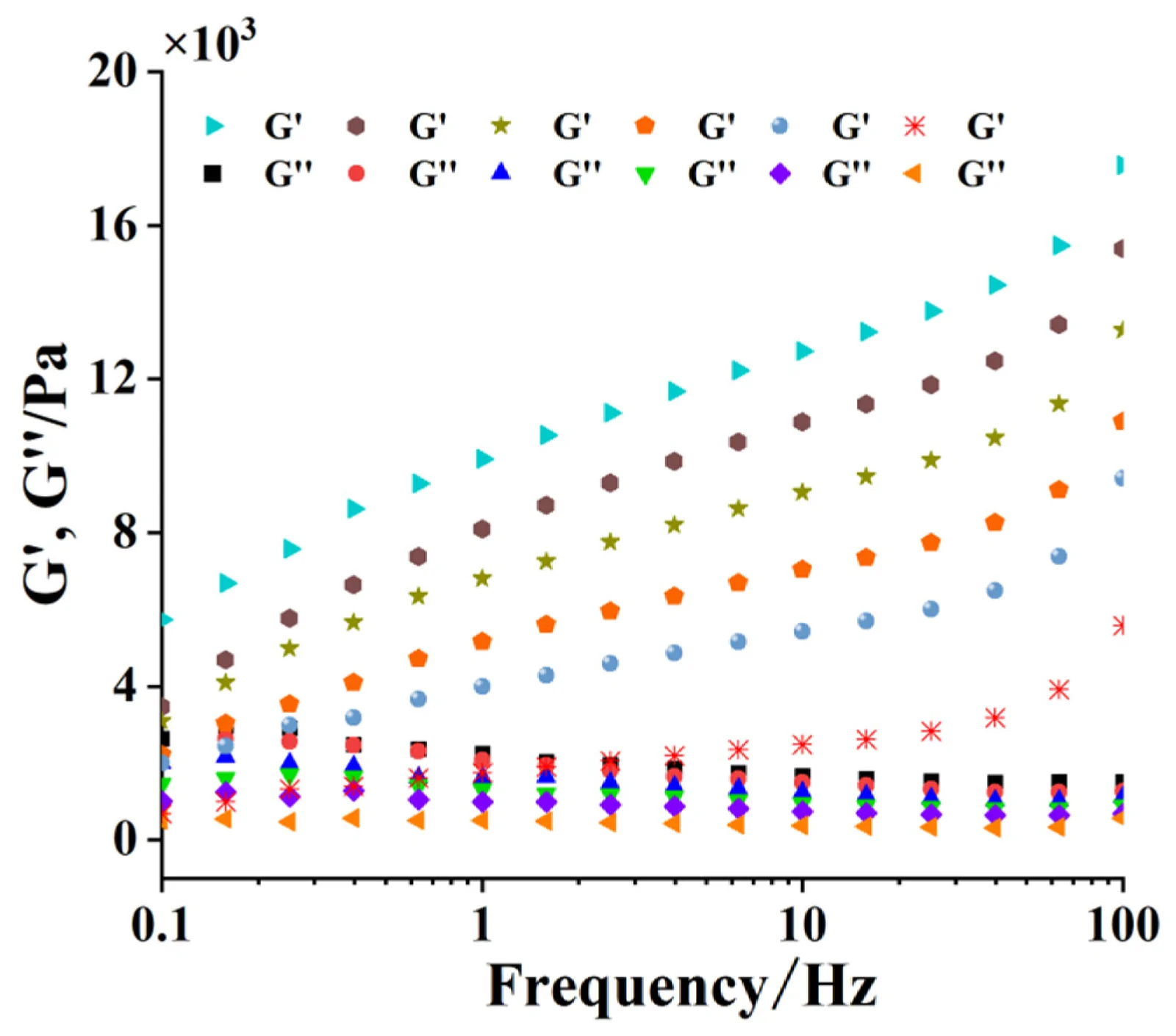
Effects of PP–HMP emulsion gel substitution level on the viscoelastic properties of whipped cream.

Figure 5 shows the effects of PP–HMP emulsion gel substitution level on the viscoelastic properties of the whipped cream. For all samples, the storage modulus (G′) increased with frequency and remained consistently higher than the loss modulus (G″). This indicated that the whipped cream samples exhibited the characteristics of an elastic system and possessed a three-dimensional gel network structure [37]. This frequency-dependent behavior was interpreted as reorganization of the fat crystal network [38]. Both G′ and G″ decreased with increasing PP–HMP emulsion gel substitution level, consistent with the hardness results of the whipped creams. This decrease might be attributed to the weakened network structure caused by the reduction in crystallizable fat globules [39]. Moreover, Farahmandfar et al. [40] reported that hydrocolloid addition modifies the network structure of whipped cream, thickening its continuous phase and forming a weak gel network through interfacial interactions with proteins. These changes improve the viscoelastic properties of whipped cream. Du and Meng [36] reported that the G′ values of creams reduce after replacing the fat phase with oleogel, indicating a weakened structural network.

**Figure 6:**
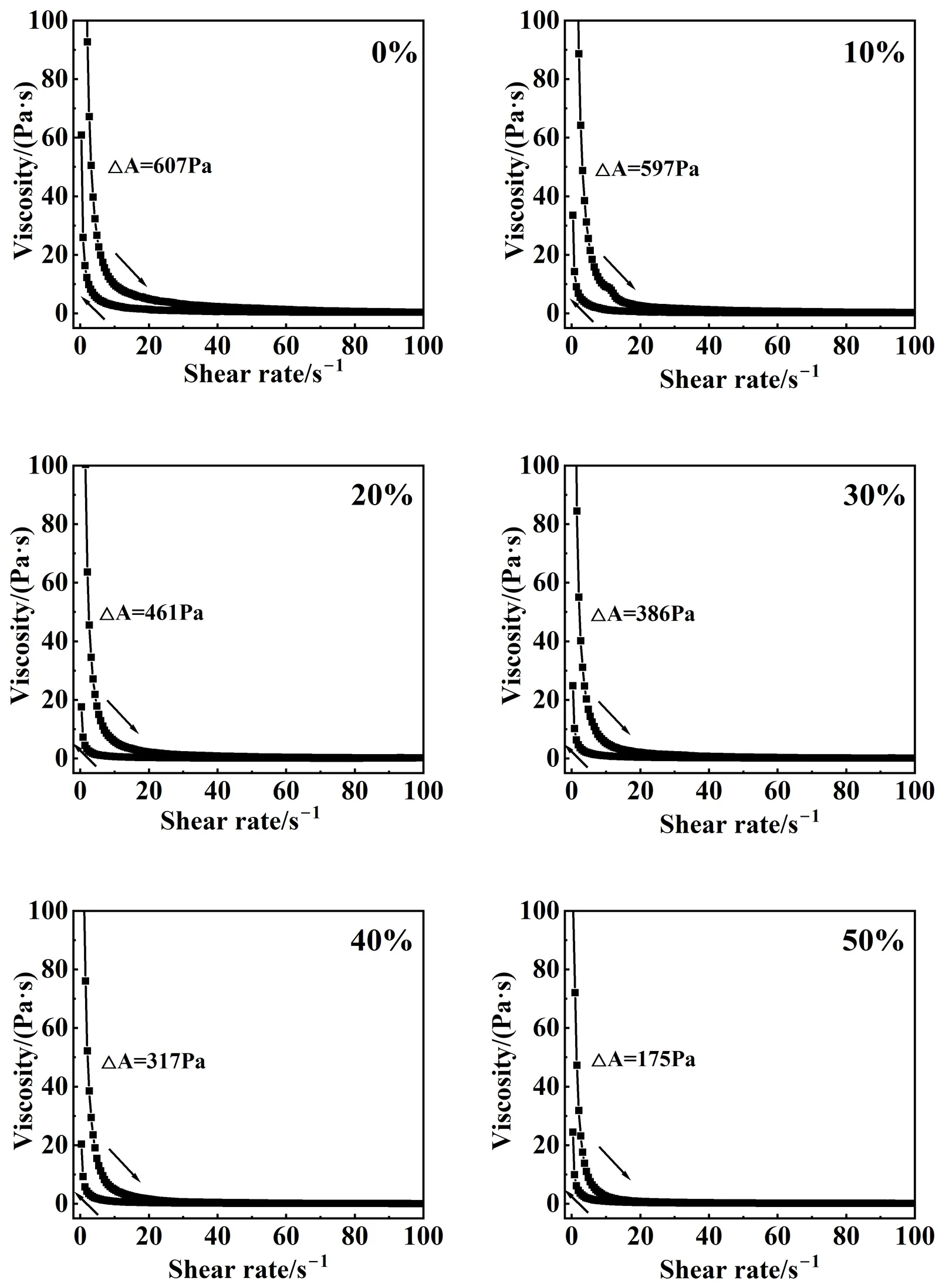
Effects of PP–HMP emulsion gel substitution level on the thixotropic behavior of whipped cream.

The shape retention ability of whipped cream primarily depends on its thixotropic properties, which can be characterized by the area of the thixotropic loop [41]. Figure 6 shows the effects of PP–HMP emulsion gel substitution level on the thixotropic properties of whipped cream. The control group exhibited the largest hysteresis loop area, and the loop area gradually decreased with increasing PP–HMP emulsion gel substitution level. None of the samples exhibited a completely closed hysteresis loop, indicating that the internal structural network of the cream could not recover fully after being subjected to shear. The decrease in the area of the thixotropic loop was related to the reduced structural recovery ability of the cream [42].

Based on the comprehensive evaluation of whipping properties, partial coalescence of fat, texture, and rheological behavior, the incorporation of PP–HMP emulsion gel containing soybean oil at a 20% substitution level provided the optimal balance between pre-whipping cream emulsion properties and whipping performance. This formulation exhibited the highest measured partial coalescence degree (48.83%) and overrun (378%) while maintaining good foam stability, shape retention, and gel network strength. In contrast, higher substitution levels (>30%) hindered partial coalescence of fat and reduced network integrity; this resulted in lower hardness, formation of larger air bubbles, increased foam collapse, and poor whipping performance. A 20% substitution level of PP–HMP emulsion gel yielded good whipping performance and shape retention. Therefore, whipped cream containing 20% PP–HMP emulsion gel was selected for performance comparison with commercial whipped cream products.

### 3.3. Comparison with Commercial Whipped Cream

#### 3.3.1. Properties of Pre-Whipping Cream Emulsions

**Figure 7:**
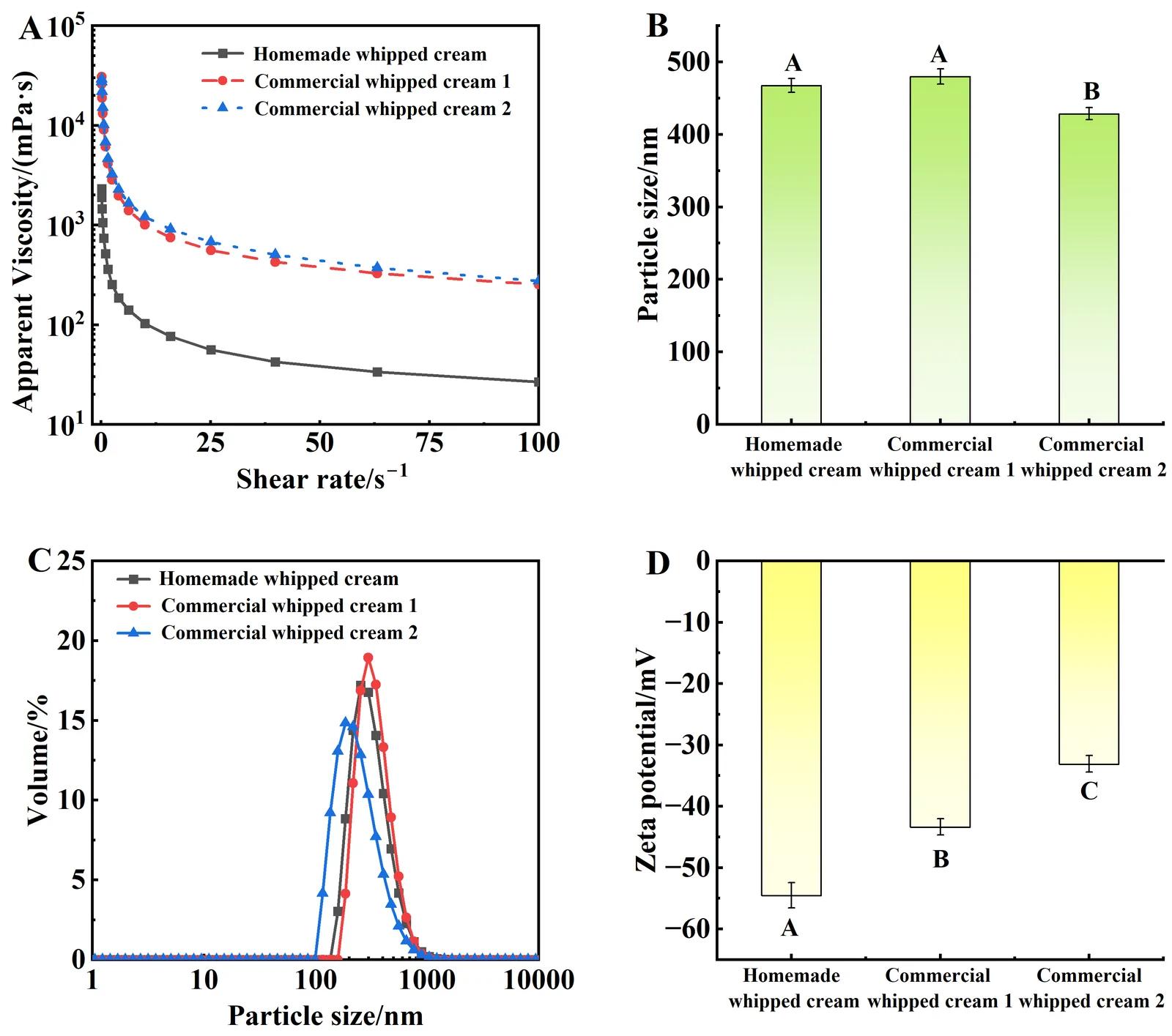
Comparison of the properties of homemade and commercial pre-whipping cream emulsions: (**A**) apparent viscosity, (**B**) particle size, (**C**) particle size distribution, and (**D**) zeta potential. Different letters indicate significant differences (*p* < 0.05).

The homemade pre-whipping cream emulsion exhibited lower apparent viscosity than the commercial pre-whipping cream emulsion (Figure 7A), but no significant differences in particle sizes were observed between the two emulsion types (Figure 7B). Figure 7C shows that both emulsions exhibited unimodal particle size distributions with smooth peaks and no satellite peaks, indicating homogeneous droplet distribution. The homemade pre-whipping cream emulsion exhibited the highest absolute zeta potential (Figure 7D). This may be attributed to the presence of PP–HMP emulsion gel, which formed a dense protein–polysaccharide interfacial layer with enhanced electrostatic repulsion [43]. This interfacial structure likely increased the negative charge density on the surface of fat globules, resulting in a high absolute zeta potential and consequently improved emulsion stability [20].

#### 3.3.2. Comparison of Whipping Characteristics

**Figure 8:**
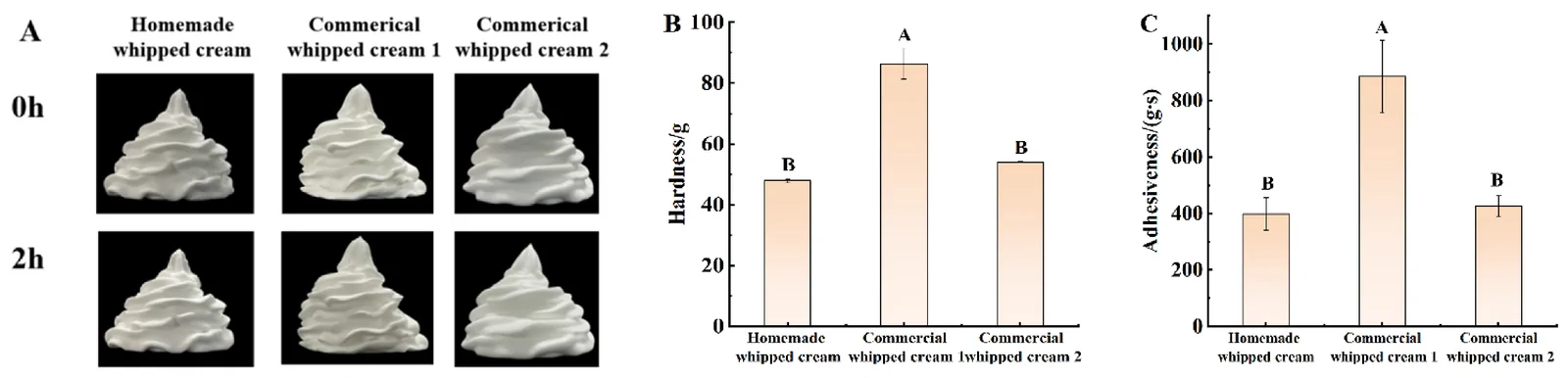
Comparison of the appearances, hardnesses and adhesiveness of homemade and commercial whipped creams: (**A**) appearance immediately after whipping and after 2 h of storage, (**B**) hardness and (**C**) adhesiveness. Different letters indicate significant differences (*p* < 0.05).

The whipping characteristics of whipped creams are shown in Table 2. The homemade whipping cream required a whipping time of 227.5 s, which was longer than that of the commercial products. The hardness and adhesiveness of the homemade whipped cream were comparable to those of commercial whipped cream 2, but lower than commercial whipped cream 1 (Figure 8). This difference is possibly attributable to the different fat composition of the creams. Being rich in lauric and myristic acids, hydrogenated palm kernel oil (commercial whipped cream 1) facilitates the formation of small crystals and dense crystal networks, promoting partial coalescence and thereby shortening the whipping time and increasing the firmness [44]. Intermolecular crosslinking of the fat molecules promotes the formation of a dense fat globule network that enhances the adhesiveness of the cream [10]. In the homemade whipped cream, the number of fat globules decreased, and the network structure became less dense, leading to decreased adhesiveness. The homemade whipped cream achieved an overrun of 378%, exceeding those of commercial whipped cream 1 (312%) and commercial whipped cream 2 (363%). After substituting hydrogenated palm kernel oil, the solid fat content decreased, and the fat crystal growth pattern shifted from two-dimensional disk-like to one-dimensional linear growth. Moderate substitution (30–60%) facilitated partial coalescence and improved overrun [11]. In addition, the homemade whipped cream exhibited a foam collapse rate of 6.85%, ranging between those of commercial whipped creams 1 and 2. The high overrun and satisfactory foam stability of the homemade whipped cream indicated that the incorporation of PP–HMP emulsion gel facilitated the formation of a stable network structure that entrapped and stabilized air bubbles [39]. The whipping performance of the homemade cream was attributable to the favorable properties of the pre-whipping emulsion. In particular, the homemade cream exhibited a particle size comparable to that of commercial cream 1 and the highest absolute zeta potential among the samples, suggesting good emulsion stability and a relatively uniform droplet size distribution. These improvements are conducive to higher overrun and satisfactory whipping performance [45]. Notably, compositional differences fundamentally limit the direct comparison of the three whipped creams. Nevertheless, the homemade whipped cream was comparable to the commercial products in terms of whipping properties.

## 4. Conclusions

Increasing the PP–HMP emulsion gel substitution level decreased the apparent viscosity and particle size of the pre-whipping cream emulsion. The partial coalescence of fat reached a maximum value of 48.83% at the 20% substitution level. Higher substitution levels reduced the hardness, adhesiveness, G′, and thixotropic loop area of the whipped cream while increasing the whipping time. A moderate substitution level (20%) provided the best balance among partial coalescence of fat, foam formation, and structural integrity, resulting in desirable whipping performance and good shape retention. Compared with commercial products, the homemade whipped cream exhibited comparable overrun, hardness, and foam stability. Substituting 20% of the cocoa butter substitute with PP–HMP emulsion gel containing soybean oil is recommended as the optimal formulation for developing a non-dairy whipping cream with satisfactory properties. Future studies should investigate the flavor, digestion, nutrient bioavailability, and metabolic effects of non-dairy whipping creams containing PP–HMP emulsion gels to further evaluate their potential health benefits.

## Figures and Tables

**Table 1:** Effects of PP–HMP emulsion gel substitution level on the partial coalescence of fat and the whipping properties of whipped cream.

Substitution Level	Partial Coalescence of Fat/%	Whipping Time/s	Overrun/%	Foam Collapse Rate/%
0%	37.18 ± 0.30 ^d^	208 ± 2.80 ^e^	375.68 ± 4.82 ^ab^	4.97 ± 0.41 ^d^
10%	40.37 ± 1.84 ^cd^	217 ± 2.10 ^de^	382.77 ± 5.47 ^a^	5.46 ± 0.36 ^d^
20%	48.83 ± 1.23 ^a^	228 ± 3.50 ^d^	378.82 ± 3.39 ^a^	6.85 ± 0.06 ^c^
30%	45.58 ± 1.74 ^ab^	253 ± 7.80 ^c^	366.37 ± 3.50 ^bc^	8.29 ± 0.18 ^b^
40%	42.40 ± 0.64 ^bc^	272 ± 4.20 ^b^	355.05 ± 7.13 ^cd^	9.25 ± 0.78 ^b^
50%	39.03 ± 2.55 ^cd^	285 ± 5.00 ^a^	350.25 ± 3.08 ^d^	10.60 ± 0.37 ^a^

Note: Different superscript letters within the same column indicate significant differences (*p* < 0.05).

**Table 2:** Comparison of whipping properties of the homemade and commercial whipped creams.

Samples	Whipping Time/s	Overrun/%	Foam Collapse Rate/%
Homemade whipped cream	227.5 ± 3.5 ^a^	378.82 ± 3.39 ^a^	6.85 ± 0.06 ^b^
Commercial whipped cream 1	146.0 ± 5.7 ^b^	312.70 ± 4.12 ^c^	5.70 ± 0.62 ^b^
Commercial whipped cream 2	125.0 ± 5.7 ^c^	363.88 ± 1.16 ^b^	10.32 ± 0.45 ^a^

Note: Different letters within the same column indicate significant differences (*p* < 0.05).

## Data Availability

The original contributions presented in this study are included in the article/Supplementary Material. Further inquiries can be directed to the corresponding authors.

## Supplementary Materials

The following supporting information can be downloaded at: https://www.mdpi.com/article/10.3390/foods15162929/s1. Table S1: The composition of commercial whipped creams; Table S2: Herschel-Bulkley fitting parameters for pre-whipping cream emulsions.

## Abbreviations

The following abbreviations are used in this manuscript:

PP	Pea protein
HMP	High-methoxyl pectin
