# Supplementary material for: Impact of Pea Protein–Pectin Emulsion Gels Containing Soybean Oil on the Whipping Performance of Non-Dairy Whipping Cream
Source: Foods. 2026 Aug 21;15(16):2929. doi: 10.3390/foods15162929 (PMC13512275; doi:10.3390/foods15162929)
Supplement: Supplementary file 1 [file foods-15-02929-s001.zip › foods-4446645-supplementary.pdf]

Table S1 The composition of commercial whipped creams

Samples	Commercial whipped cream 1	Commercial whipped cream 2
Main components	Water, Hydrogenated palm kernel oil, Glucose, Glucose syrup, White sugar	Water, Maltose syrup, Hydrogenated vegetable oil, Glucose, White sugar
Emulsifier	Sodium caseinate, Sodium stearoyl lactylate, Polyglycerol fatty acid esters, Sucrose fatty acid esters, Mono- and diglycerides of fatty acids, Span80	Sodium stearoyl lactylate, Sodium caseinate, Span80, Sorbitan monostearate, Mono- and diglycerides of fatty acids, Phospholipid, Polyglycerol fatty acid esters, Sorbitan monostearate
Thickener	Hydroxypropyl methylcellulose, Xanthan gum, Carrageenan, Locust bean gum	Guar gum, Xanthan gum, Carrageenan, Hydroxypropyl methylcellulose
Moisture Retainer	Dipotassium hydrogen phosphate, Sodium pyrophosphate, Sorbitol solution	Dipotassium hydrogen phosphate
Others	Salt, Food flavor, Coloring agent	Sweetener, Salt, Food flavor

Table S2. Herschel-Bulkley fitting parameters for pre-whipping cream emulsions

**Substitution level**	**Yield stress (τ_0)(Pa)**	**Consistency coefficient (*K*) (Pa·s^n)**	**Flow index (*n*)**
0%	0.13	0.09	0.76
10%	0.20	0.12	0.69
20%	0.30	0.17	0.58
30%	0.29	0.11	0.65
40%	0.25	0.08	0.68
50%	0.25	0.09	0.60
